# Crystal structure of sepaconitine, a C_19_-diterpenoid alkaloid from the roots of *Aconitum sinomontanum* Nakai

**DOI:** 10.1107/S205698901501258X

**Published:** 2015-07-08

**Authors:** Xin-Wei Shi, Qiang-Qiang Lu, Jun-Hui Zhou, Xin-Ai Cui

**Affiliations:** a Xi’an Botanical Garden, Institute of Botany of Shaanxi Province, Xi’an 710061, People’s Republic of China

**Keywords:** crystal structure, C_19_-diterpenoid alkaloid, hydrogen bonding

## Abstract

The title compound [systematic name: [(1α,14α,16β)-20-ethyl-8,9,10-trihy­droxy-1,14,16-tri­meth­oxy­aconitan-4-yl 2-amino­benzoate], C_30_H_42_N_2_O_8_, a natural C_19_-diterpenoid alkaloid, possesses an aconitane carbon skeleton with four six-membered rings and two five-membered rings. The fused ring system contains two chair, one boat, one twist-boat and two envelope conformations. Intra­molecular N—H⋯O hydrogen bonds are observed between the amino and carbonyl groups. The mol­ecules are linked together *via* O—H⋯O hydrogen bonds, forming a three-dimensional framework.

## Related literature   

For the synthesis of the title compound, see: Wei *et al.* (1996 ▸). The absolute configuration of the title compound has been assigned to be the same as that reported for typical natural C_19_-diterpenoid alkaloids, see: Wang *et al.* (2007 ▸); He *et al.* (2008 ▸). The six-ring rigid-frame structure of the title compound is identical to that of lappaconitine and mesaconitine (Wang *et al.*, 2007 ▸; He *et al.*, 2008 ▸).
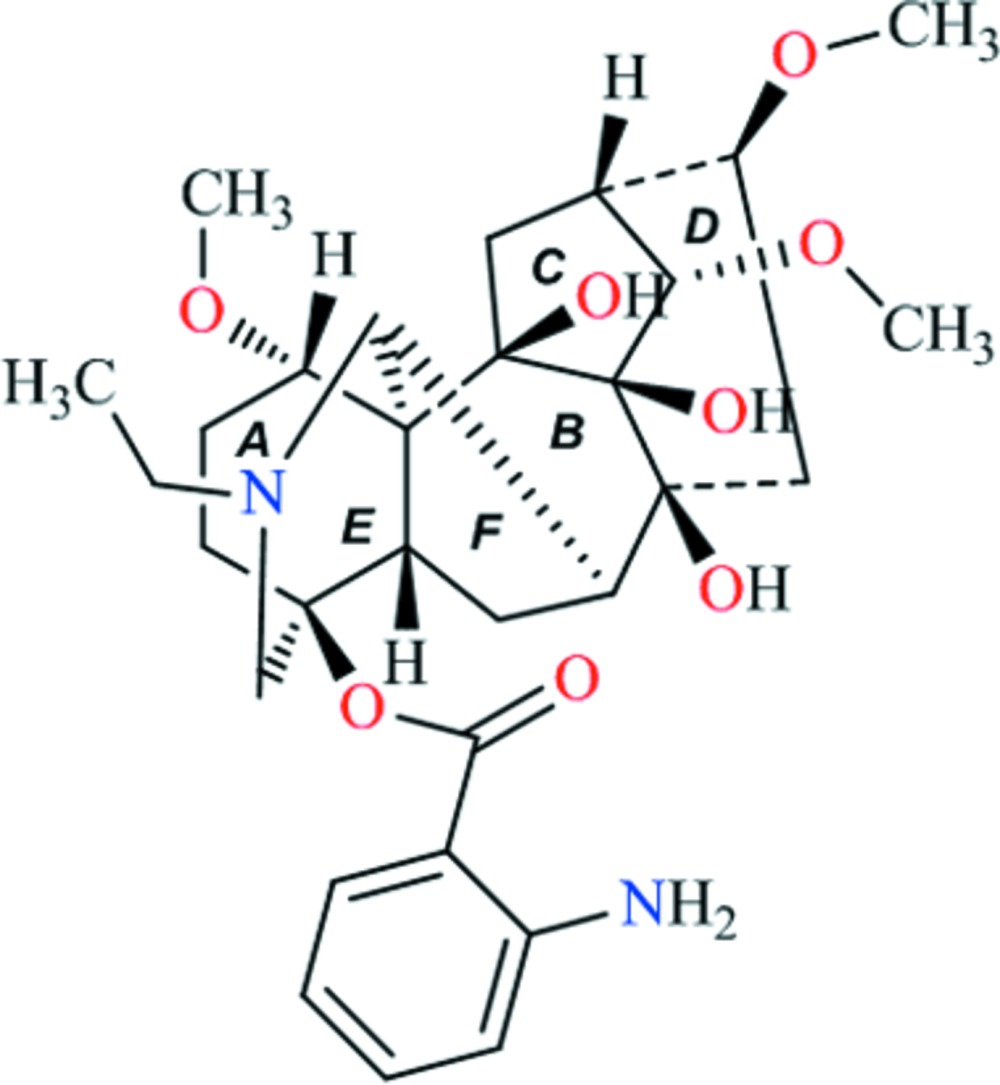


## Experimental   

### Crystal data  

C_30_H_42_N_2_O_8_
*M* *_r_* = 558.66
Orthorhombic, 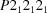

*a* = 9.6917 (5) Å
*b* = 16.0510 (7) Å
*c* = 18.3549 (7) Å
*V* = 2855.3 (2) Å^3^
*Z* = 4
Cu *K*α radiation
μ = 0.77 mm^−1^
*T* = 173 K
0.32 × 0.32 × 0.28 mm

### Data collection  

Bruker SMART CCD area-detector diffractometer
Absorption correction: multi-scan (*SADABS*; Bruker, 2002 ▸) *T*_min_ = 0.791, *T*_max_ = 0.813
8125 measured reflections
4528 independent reflections
3831 reflections with *I* > 2σ(*I*)
*R*_int_ = 0.029

### Refinement  

*R*[*F* ^2^ > 2σ(*F* ^2^)] = 0.046
*wR*(*F* ^2^) = 0.126
*S* = 1.05
4528 reflections
388 parameters
60 restraints
H-atom parameters constrained
Δρ_max_ = 0.46 e Å^−3^
Δρ_min_ = −0.19 e Å^−3^

### 

Data collection: *SMART* (Bruker, 2002 ▸); cell refinement: *SAINT* (Bruker, 2002 ▸); data reduction: *SAINT*; program(s) used to solve structure: *SHELXTL* (Sheldrick, 2008 ▸); program(s) used to refine structure: *SHELXTL*; molecular graphics: *SHELXTL*; software used to prepare material for publication: *SHELXTL*.

## Supplementary Material

Crystal structure: contains datablock(s) I, New_Global_Publ_Block. DOI: 10.1107/S205698901501258X/hg5447sup1.cif

Structure factors: contains datablock(s) I. DOI: 10.1107/S205698901501258X/hg5447Isup2.hkl

Supporting information file. DOI: 10.1107/S205698901501258X/hg5447Isup4.cdx

**Fig. 1.**
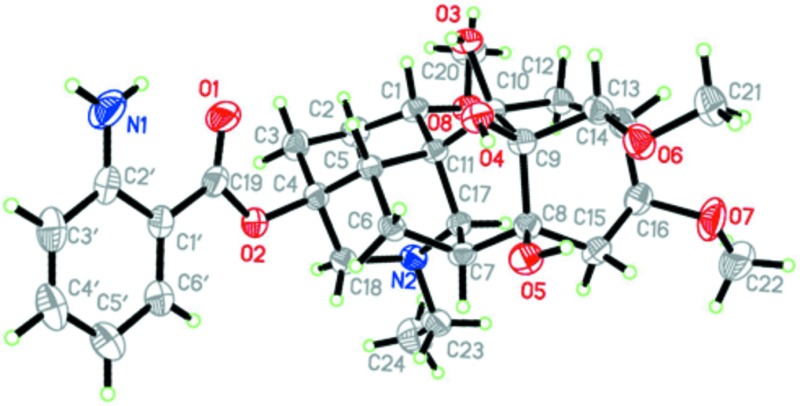
ORTEPII drawing of sepaconitine (I) with the atomic numbering scheme. Displacement ellipsoids are plotted at the 50% probability level.

**Fig. 2.**
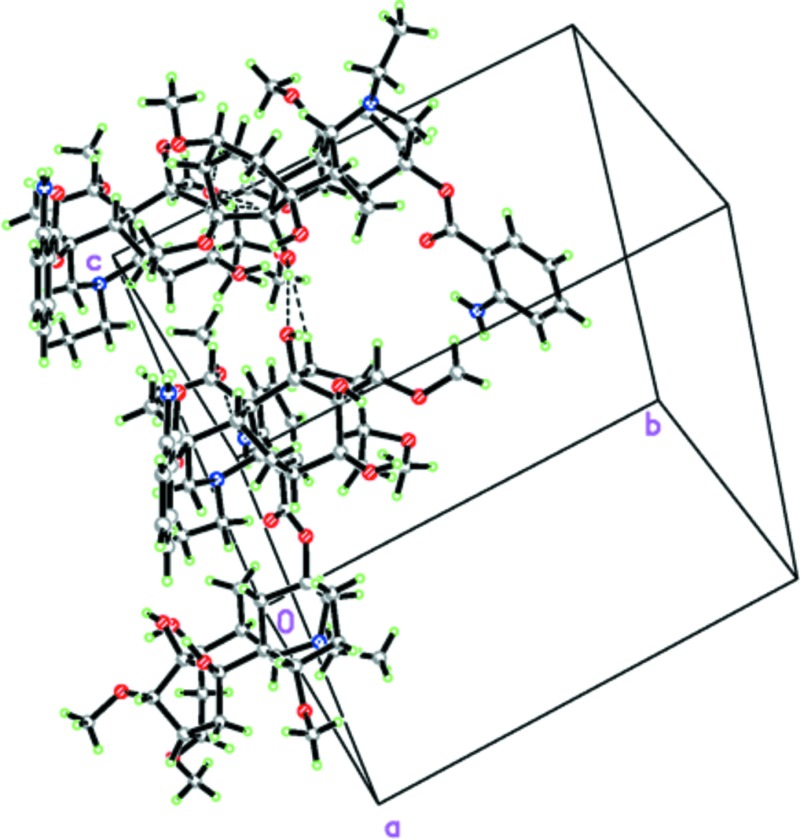
The packing of molecules in the crystal structure of sepaconitine (I), viewed along the c direction (Hydrogen bonds are shown as dashed lines).

ORTEPII . DOI: 10.1107/S205698901501258X/hg5447fig1.tif
*ORTEPII* drawing of sepaconitine (I) with the atomic numbering scheme. Displacement ellipsoids are plotted at the 50% probability level.

c . DOI: 10.1107/S205698901501258X/hg5447fig2.tif
The packing of mol­ecules in the crystal structure of sepaconitine (I), viewed along the *c* direction (Hydrogen bonds are shown as dashed lines).

CCDC reference: 1409635

Additional supporting information:  crystallographic information; 3D view; checkCIF report

## Figures and Tables

**Table 1:** Hydrogen-bond geometry (, )

*D*H*A*	*D*H	H*A*	*D* *A*	*D*H*A*
O5H5*A*O6	0.84	2.34	2.909(3)	126
O4H4O5	0.84	2.27	2.684(3)	111
O4H4O3^i^	0.84	1.94	2.713(3)	153
O3H3O4	0.84	1.99	2.524(3)	121
N1H1*B*O1	0.88	2.00	2.666(4)	131
N1H1*A*O8^ii^	0.88	2.19	2.999(3)	152

Symmetry codes: (i) 
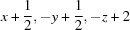
; (ii) 
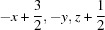
.

## supplementary crystallographic information

### S1. Comment

The title compound was isolated from the roots of *Aconitum sinomontanum* Nakai, collected in Taibai mountain of the Qinling area, Shaanxi province, People's Republic of China. the crystal structure determination of sepaconitine was carried out and the result reported here.

The molecular structure is shown in Fig. 1. The molecule has a rigid structure consisting of six main rings (*A*—*F*), which is identical with that of lappaconitine and mesaconitine (Wang *et al.*, 2007; He *et al.*, 2008). The six-membered rings *A* (C1/C2/C3/C4/C5/C11) and N-containing heterocyclic ring *E* (C4/C5/C11/C17/N2/C18) adopt chair conformations; The six-membered ring *D* (C8/C9/C14/C13/C16/C15) displays a boat conformation, but *B* (C7/C8/C9/C10/C11/C17) adopts a twist-boat conformation; the five-membered rings *C* (C9/C10/C12/C13/C14) and *F* (C5/C6/C7/C17/C11) adopt C14- and C 17-envelope conformations, respectively. Two *cis*-fused ring junctions involve rings *A*/*E* and also *B*/*C*. Two *trans*-fused ring junctions are observed between rings *A*/*B* and between *E*/*F*. Ring *E* is slightly flattened at C5 due to the presence of an ethyl-substituted N atom in the ring. The benzoate moiety attached to C4 is almost planar. The OCH_3_ group attached to C16 is disordered into two positions with site occupancies Factor of 0.5.

The crystal structure has an intra-molecular N—H···O hydrogen bond between the amino group and carbonyl O atom (Table 1). Inter-molecular N—H···O hydrogen bonds are observed in the crystal. The molecules are linked together via O—H```O hydrogen bonds in the *c* direction (Table 1, Fig. 2).

### S2. Experimental

The title compound was isolated from the roots of *Aconitum sinomontanum*, using a method described previously (Wei *et al.*, 1996). Colourless crystals were grown from methanol at room temperature by slow evaporation.

### S3. Refinement

The hydrogen atoms were placed in calculated positions and refined as riding with U_iso_(H) = 1.2 Ueq (C) or 1.5Ueq(C, O). The positions of methyl and hy­droxy hydrogens were rotationally optimized. The absolute configuration of the title compound, sepaconitine, has been assigned to be the same as that reported for typical natural C_19_-diterpenoid alkaloids (Wang *et al.*, 2007; He *et al.*, 2008).

### Crystal data

**Table:**

C_30_H_42_N_2_O_8_	*D*_x_ = 1.300 Mg m^−^^3^
*M**_r_* = 558.66	Melting point = 250–252 K
Orthorhombic, *P*2_1_2_1_2_1_	Cu *K*α radiation, λ = 1.54178 Å
Hall symbol: P 2ac 2ab	Cell parameters from 2628 reflections
*a* = 9.6917 (5) Å	θ = 3.7–64.2°
*b* = 16.0510 (7) Å	µ = 0.77 mm^−^^1^
*c* = 18.3549 (7) Å	*T* = 173 K
*V* = 2855.3 (2) Å^3^	Block, colorless
*Z* = 4	0.32 × 0.32 × 0.28 mm
*F*(000) = 1200	

### Data collection

**Table:**

Bruker SMART CCD area-detector diffractometer	4528 independent reflections
Radiation source: fine-focus sealed tube	3831 reflections with *I* > 2σ(*I*)
Graphite monochromator	*R*_int_ = 0.029
phi and ω scans	θ_max_ = 65.0°, θ_min_ = 3.7°
Absorption correction: multi-scan (*SADABS*; Bruker, 2002)	*h* = −7→11
*T*_min_ = 0.791, *T*_max_ = 0.813	*k* = −18→18
8125 measured reflections	*l* = −21→18

### Refinement

**Table:**

Refinement on *F*^2^	Primary atom site location: structure-invariant direct methods
Least-squares matrix: full	Secondary atom site location: difference Fourier map
*R*[*F*^2^ > 2σ(*F*^2^)] = 0.046	Hydrogen site location: inferred from neighbouring sites
*wR*(*F*^2^) = 0.126	H-atom parameters constrained
*S* = 1.05	*w* = 1/[σ^2^(*F*_o_^2^) + (0.0752*P*)^2^ + 0.0411*P*] where *P* = (*F*_o_^2^ + 2*F*_c_^2^)/3
4528 reflections	(Δ/σ)_max_ = 0.001
388 parameters	Δρ_max_ = 0.46 e Å^−^^3^
60 restraints	Δρ_min_ = −0.19 e Å^−^^3^

### Special details

**Table:**

Geometry. All e.s.d.'s (except the e.s.d. in the dihedral angle between two l.s. planes) are estimated using the full covariance matrix. The cell e.s.d.'s are taken into account individually in the estimation of e.s.d.'s in distances, angles and torsion angles; correlations between e.s.d.'s in cell parameters are only used when they are defined by crystal symmetry. An approximate (isotropic) treatment of cell e.s.d.'s is used for estimating e.s.d.'s involving l.s. planes.
Refinement. Refinement of *F*^2^ against ALL reflections. The weighted *R*-factor *wR* and goodness of fit *S* are based on *F*^2^, conventional *R*-factors *R* are based on *F*, with *F* set to zero for negative *F*^2^. The threshold expression of *F*^2^ > σ(*F*^2^) is used only for calculating *R*-factors(gt) *etc*. and is not relevant to the choice of reflections for refinement. *R*-factors based on *F*^2^ are statistically about twice as large as those based on *F*, and *R*- factors based on ALL data will be even larger.

### Fractional atomic coordinates and isotropic or equivalent isotropic displacement parameters (Å^2^)

**Table:**

	*x*	*y*	*z*	*U*_iso_*/*U*_eq_	Occ. (<1)
C1	0.5519 (3)	0.04441 (18)	0.93627 (14)	0.0459 (6)	
H1	0.5224	0.0713	0.9829	0.055*	
C2	0.5401 (3)	−0.04952 (19)	0.94729 (17)	0.0574 (7)	
H2A	0.4494	−0.0626	0.9693	0.069*	
H2B	0.5451	−0.0777	0.8994	0.069*	
C3	0.6545 (3)	−0.08242 (19)	0.99638 (17)	0.0587 (7)	
H3A	0.6440	−0.0598	1.0462	0.070*	
H3B	0.6503	−0.1440	0.9990	0.070*	
C4	0.7912 (3)	−0.05507 (17)	0.96421 (14)	0.0472 (6)	
C5	0.8106 (3)	0.03991 (16)	0.96718 (13)	0.0417 (6)	
H5	0.8054	0.0615	1.0182	0.050*	
C6	0.9497 (3)	0.06134 (17)	0.93102 (14)	0.0457 (6)	
H6A	1.0101	0.0117	0.9287	0.055*	
H6B	0.9979	0.1059	0.9583	0.055*	
C7	0.9109 (3)	0.09122 (16)	0.85379 (13)	0.0443 (6)	
H7	0.9740	0.0659	0.8168	0.053*	
C8	0.9157 (3)	0.18622 (16)	0.84910 (14)	0.0445 (6)	
C9	0.8267 (3)	0.22508 (16)	0.91195 (13)	0.0416 (6)	
C10	0.6911 (3)	0.17703 (16)	0.92456 (12)	0.0405 (6)	
C11	0.6968 (3)	0.07934 (16)	0.91761 (13)	0.0399 (5)	
C12	0.5904 (3)	0.22027 (18)	0.87023 (15)	0.0496 (6)	
H12A	0.5568	0.1796	0.8338	0.060*	
H12B	0.5098	0.2431	0.8967	0.060*	
C13	0.6700 (3)	0.29100 (18)	0.83222 (15)	0.0527 (7)	
H13	0.6084	0.3399	0.8230	0.063*	
C14	0.7783 (3)	0.31184 (17)	0.88965 (15)	0.0512 (7)	
H14	0.7319	0.3389	0.9322	0.061*	
C15	0.8737 (3)	0.2179 (2)	0.77238 (14)	0.0565 (7)	
H15A	0.9459	0.2574	0.7560	0.068*	
H15B	0.8767	0.1696	0.7389	0.068*	
C16	0.7352 (4)	0.2605 (2)	0.76183 (15)	0.0604 (8)	
H16	0.6702	0.2225	0.7356	0.073*	
C17	0.7620 (3)	0.05823 (16)	0.84348 (12)	0.0415 (6)	
H17	0.7146	0.0900	0.8039	0.050*	
C18	0.8127 (3)	−0.08526 (17)	0.88536 (14)	0.0515 (7)	
H18A	0.9130	−0.0893	0.8758	0.062*	
H18B	0.7734	−0.1419	0.8806	0.062*	
C19	0.9371 (3)	−0.08098 (19)	1.07202 (15)	0.0556 (7)	
C20	0.3182 (3)	0.0800 (3)	0.9099 (2)	0.0738 (10)	
H20A	0.2839	0.0276	0.9305	0.111*	
H20B	0.2567	0.0982	0.8706	0.111*	
H20C	0.3209	0.1227	0.9480	0.111*	
C21	0.8619 (4)	0.4468 (2)	0.8583 (2)	0.0807 (10)	
H21A	0.8080	0.4534	0.8135	0.121*	
H21B	0.9477	0.4788	0.8543	0.121*	
H21C	0.8081	0.4672	0.8999	0.121*	
C22	0.7499 (12)	0.3113 (7)	0.6432 (4)	0.114 (3)	0.578 (12)
H22A	0.8162	0.2697	0.6260	0.170*	0.578 (12)
H22B	0.7575	0.3617	0.6134	0.170*	0.578 (12)
H22C	0.6561	0.2888	0.6393	0.170*	0.578 (12)
C22'	0.6743 (13)	0.3573 (9)	0.6707 (7)	0.104 (4)	0.422 (12)
H22D	0.6386	0.3124	0.6398	0.155*	0.422 (12)
H22E	0.7364	0.3927	0.6421	0.155*	0.422 (12)
H22F	0.5972	0.3909	0.6890	0.155*	0.422 (12)
C23	0.7964 (4)	−0.0559 (2)	0.75690 (15)	0.0625 (8)	
H23A	0.8964	−0.0683	0.7583	0.075*	
H23B	0.7822	−0.0086	0.7230	0.075*	
C24	0.7206 (6)	−0.1305 (2)	0.7287 (2)	0.0994 (14)	
H24A	0.7516	−0.1803	0.7548	0.149*	
H24B	0.7394	−0.1371	0.6765	0.149*	
H24C	0.6213	−0.1229	0.7362	0.149*	
C1'	1.0692 (3)	−0.11619 (17)	1.09396 (15)	0.0525 (7)	
C2'	1.1153 (4)	−0.10849 (19)	1.16724 (16)	0.0600 (8)	
C3'	1.2446 (4)	−0.1426 (2)	1.1845 (2)	0.0732 (10)	
H3'	1.2769	−0.1389	1.2333	0.088*	
C4'	1.3246 (4)	−0.1804 (2)	1.1341 (2)	0.0786 (11)	
H4'	1.4116	−0.2025	1.1480	0.094*	
C5'	1.2809 (4)	−0.1874 (2)	1.0619 (2)	0.0739 (9)	
H5'	1.3379	−0.2134	1.0265	0.089*	
C6'	1.1544 (4)	−0.15597 (19)	1.04293 (17)	0.0624 (8)	
H6'	1.1236	−0.1613	0.9940	0.075*	
N1	1.0397 (4)	−0.0685 (2)	1.21841 (14)	0.0858 (10)	
H1A	1.0716	−0.0636	1.2631	0.103*	
H1B	0.9588	−0.0473	1.2070	0.103*	
N2	0.7503 (2)	−0.03157 (13)	0.82988 (10)	0.0461 (5)	
O1	0.8590 (3)	−0.04165 (18)	1.11148 (11)	0.0792 (7)	
O2	0.9070 (2)	−0.09711 (12)	1.00172 (10)	0.0533 (5)	
O3	0.64582 (19)	0.19706 (12)	0.99729 (9)	0.0492 (4)	
H3	0.7146	0.2082	1.0234	0.074*	
O4	0.8988 (2)	0.22811 (12)	0.97916 (9)	0.0493 (5)	
H4	0.9790	0.2463	0.9721	0.074*	
O5	1.05780 (19)	0.20907 (13)	0.86056 (11)	0.0567 (5)	
H5A	1.0672	0.2605	0.8540	0.085*	
O6	0.8938 (2)	0.36055 (12)	0.86885 (12)	0.0613 (5)	
O7	0.7770 (13)	0.3303 (12)	0.7134 (9)	0.092 (3)	0.578 (12)
O7'	0.7307 (16)	0.3306 (16)	0.7166 (13)	0.084 (3)	0.422 (12)
O8	0.45273 (18)	0.06771 (13)	0.88177 (10)	0.0525 (5)	

### Atomic displacement parameters (Å^2^)

**Table:**

	*U*^11^	*U*^22^	*U*^33^	*U*^12^	*U*^13^	*U*^23^
C1	0.0340 (14)	0.0631 (16)	0.0404 (12)	−0.0009 (13)	0.0003 (11)	−0.0027 (11)
C2	0.0407 (16)	0.0666 (18)	0.0650 (17)	−0.0101 (15)	0.0019 (13)	0.0052 (14)
C3	0.0552 (19)	0.0600 (16)	0.0609 (17)	−0.0055 (15)	0.0027 (15)	0.0118 (13)
C4	0.0425 (15)	0.0532 (15)	0.0457 (14)	0.0050 (13)	−0.0030 (11)	0.0054 (11)
C5	0.0368 (14)	0.0518 (14)	0.0365 (12)	0.0057 (12)	−0.0025 (10)	0.0001 (10)
C6	0.0378 (14)	0.0488 (14)	0.0506 (14)	0.0084 (12)	−0.0013 (12)	0.0003 (11)
C7	0.0367 (14)	0.0537 (14)	0.0424 (13)	0.0037 (12)	0.0040 (11)	−0.0013 (11)
C8	0.0348 (14)	0.0524 (14)	0.0462 (13)	−0.0005 (12)	0.0019 (11)	0.0017 (11)
C9	0.0351 (13)	0.0507 (13)	0.0390 (12)	0.0015 (12)	−0.0043 (10)	−0.0035 (10)
C10	0.0356 (14)	0.0515 (14)	0.0344 (12)	0.0059 (11)	−0.0004 (10)	−0.0047 (10)
C11	0.0297 (13)	0.0502 (13)	0.0399 (12)	−0.0002 (11)	0.0002 (10)	−0.0014 (10)
C12	0.0364 (14)	0.0590 (16)	0.0535 (15)	0.0064 (13)	−0.0080 (12)	−0.0011 (12)
C13	0.0502 (17)	0.0558 (15)	0.0522 (15)	0.0080 (14)	−0.0063 (13)	0.0050 (11)
C14	0.0480 (16)	0.0524 (15)	0.0532 (15)	0.0039 (13)	−0.0008 (13)	−0.0009 (12)
C15	0.0641 (19)	0.0610 (16)	0.0442 (14)	−0.0007 (15)	0.0094 (13)	0.0038 (12)
C16	0.069 (2)	0.0665 (18)	0.0457 (14)	0.0031 (16)	−0.0044 (14)	0.0074 (13)
C17	0.0373 (14)	0.0496 (14)	0.0376 (12)	0.0042 (12)	−0.0020 (10)	0.0008 (10)
C18	0.0553 (17)	0.0493 (14)	0.0499 (14)	0.0053 (13)	0.0003 (13)	−0.0020 (11)
C19	0.0615 (19)	0.0633 (17)	0.0420 (14)	0.0028 (16)	−0.0019 (14)	0.0099 (12)
C20	0.0339 (16)	0.106 (3)	0.082 (2)	0.0081 (18)	0.0051 (15)	−0.0039 (19)
C21	0.101 (3)	0.0491 (16)	0.092 (2)	0.0004 (19)	0.003 (2)	0.0063 (16)
C22	0.135 (6)	0.138 (6)	0.067 (4)	0.031 (5)	0.013 (4)	0.034 (4)
C22'	0.097 (6)	0.126 (6)	0.088 (6)	0.041 (5)	0.003 (5)	0.037 (5)
C23	0.072 (2)	0.0711 (19)	0.0441 (14)	0.0178 (17)	−0.0071 (14)	−0.0102 (13)
C24	0.149 (4)	0.077 (2)	0.072 (2)	0.017 (3)	−0.021 (3)	−0.0308 (19)
C1'	0.0545 (17)	0.0534 (15)	0.0496 (14)	−0.0008 (14)	−0.0054 (13)	0.0126 (12)
C2'	0.067 (2)	0.0612 (17)	0.0519 (16)	−0.0053 (16)	−0.0100 (15)	0.0187 (13)
C3'	0.080 (3)	0.069 (2)	0.071 (2)	−0.004 (2)	−0.031 (2)	0.0131 (17)
C4'	0.069 (2)	0.0639 (19)	0.103 (3)	0.0035 (19)	−0.032 (2)	0.0146 (19)
C5'	0.061 (2)	0.069 (2)	0.091 (2)	0.0112 (18)	−0.0127 (18)	0.0022 (17)
C6'	0.064 (2)	0.0612 (17)	0.0618 (18)	0.0054 (17)	−0.0093 (16)	0.0075 (14)
N1	0.089 (2)	0.125 (3)	0.0431 (13)	0.005 (2)	−0.0066 (14)	0.0039 (15)
N2	0.0471 (13)	0.0501 (12)	0.0412 (11)	0.0055 (11)	−0.0047 (10)	−0.0058 (8)
O1	0.0785 (16)	0.1133 (18)	0.0457 (11)	0.0293 (15)	−0.0017 (11)	−0.0006 (11)
O2	0.0560 (12)	0.0576 (11)	0.0462 (10)	0.0098 (9)	−0.0034 (9)	0.0067 (8)
O3	0.0427 (11)	0.0645 (11)	0.0404 (9)	0.0048 (9)	0.0031 (8)	−0.0097 (8)
O4	0.0418 (11)	0.0626 (11)	0.0436 (9)	−0.0016 (9)	−0.0073 (8)	−0.0034 (8)
O5	0.0392 (11)	0.0613 (11)	0.0695 (12)	−0.0060 (9)	0.0066 (9)	0.0040 (10)
O6	0.0618 (13)	0.0485 (10)	0.0737 (13)	−0.0011 (10)	0.0018 (10)	0.0024 (9)
O7	0.104 (6)	0.104 (3)	0.069 (3)	0.011 (6)	−0.007 (5)	0.047 (3)
O7'	0.087 (7)	0.096 (4)	0.067 (4)	0.013 (6)	0.001 (6)	0.036 (3)
O8	0.0320 (10)	0.0767 (12)	0.0488 (10)	0.0033 (9)	−0.0025 (8)	−0.0018 (9)

### Geometric parameters (Å, º)

**Table:**

C1—O8	1.437 (3)	C17—H17	1.0000
C1—C2	1.525 (4)	C18—N2	1.465 (3)
C1—C11	1.551 (3)	C18—H18A	0.9900
C1—H1	1.0000	C18—H18B	0.9900
C2—C3	1.523 (4)	C19—O1	1.223 (4)
C2—H2A	0.9900	C19—O2	1.348 (3)
C2—H2B	0.9900	C19—C1'	1.456 (4)
C3—C4	1.515 (4)	C20—O8	1.416 (3)
C3—H3A	0.9900	C20—H20A	0.9800
C3—H3B	0.9900	C20—H20B	0.9800
C4—O2	1.479 (3)	C20—H20C	0.9800
C4—C5	1.537 (4)	C21—O6	1.431 (4)
C4—C18	1.540 (4)	C21—H21A	0.9800
C5—C6	1.542 (4)	C21—H21B	0.9800
C5—C11	1.563 (3)	C21—H21C	0.9800
C5—H5	1.0000	C22—O7	1.350 (19)
C6—C7	1.543 (3)	C22—H22A	0.9800
C6—H6A	0.9900	C22—H22B	0.9800
C6—H6B	0.9900	C22—H22C	0.9800
C7—C8	1.528 (4)	C22'—O7'	1.09 (2)
C7—C17	1.549 (4)	C22'—H22D	0.9800
C7—H7	1.0000	C22'—H22E	0.9800
C8—O5	1.440 (3)	C22'—H22F	0.9800
C8—C15	1.552 (4)	C23—N2	1.465 (3)
C8—C9	1.570 (4)	C23—C24	1.497 (5)
C9—O4	1.419 (3)	C23—H23A	0.9900
C9—C14	1.525 (4)	C23—H23B	0.9900
C9—C10	1.542 (4)	C24—H24A	0.9800
C10—O3	1.442 (3)	C24—H24B	0.9800
C10—C12	1.558 (3)	C24—H24C	0.9800
C10—C11	1.574 (4)	C1'—C6'	1.402 (4)
C11—C17	1.538 (3)	C1'—C2'	1.423 (4)
C12—C13	1.540 (4)	C2'—N1	1.353 (4)
C12—H12A	0.9900	C2'—C3'	1.404 (5)
C12—H12B	0.9900	C3'—C4'	1.352 (5)
C13—C16	1.520 (4)	C3'—H3'	0.9500
C13—C14	1.525 (4)	C4'—C5'	1.397 (5)
C13—H13	1.0000	C4'—H4'	0.9500
C14—O6	1.418 (4)	C5'—C6'	1.371 (5)
C14—H14	1.0000	C5'—H5'	0.9500
C15—C16	1.518 (5)	C6'—H6'	0.9500
C15—H15A	0.9900	N1—H1A	0.8800
C15—H15B	0.9900	N1—H1B	0.8800
C16—O7'	1.40 (2)	O3—H3	0.8400
C16—O7	1.487 (17)	O4—H4	0.8400
C16—H16	1.0000	O5—H5A	0.8400
C17—N2	1.467 (3)		
			
O8—C1—C2	107.4 (2)	C8—C15—H15B	107.4
O8—C1—C11	111.0 (2)	H15A—C15—H15B	106.9
C2—C1—C11	117.0 (2)	O7'—C16—C15	117.7 (7)
O8—C1—H1	107.0	O7—C16—C15	100.0 (5)
C2—C1—H1	107.0	O7'—C16—C13	103.4 (9)
C11—C1—H1	107.0	O7—C16—C13	112.2 (7)
C3—C2—C1	111.5 (2)	C15—C16—C13	113.8 (2)
C3—C2—H2A	109.3	O7'—C16—H16	100.7
C1—C2—H2A	109.3	O7—C16—H16	110.1
C3—C2—H2B	109.3	C15—C16—H16	110.1
C1—C2—H2B	109.3	C13—C16—H16	110.1
H2A—C2—H2B	108.0	N2—C17—C11	109.6 (2)
C4—C3—C2	107.8 (2)	N2—C17—C7	115.4 (2)
C4—C3—H3A	110.1	C11—C17—C7	101.48 (18)
C2—C3—H3A	110.1	N2—C17—H17	110.0
C4—C3—H3B	110.1	C11—C17—H17	110.0
C2—C3—H3B	110.1	C7—C17—H17	110.0
H3A—C3—H3B	108.5	N2—C18—C4	114.3 (2)
O2—C4—C3	110.5 (2)	N2—C18—H18A	108.7
O2—C4—C5	110.1 (2)	C4—C18—H18A	108.7
C3—C4—C5	112.4 (2)	N2—C18—H18B	108.7
O2—C4—C18	101.0 (2)	C4—C18—H18B	108.7
C3—C4—C18	113.2 (2)	H18A—C18—H18B	107.6
C5—C4—C18	109.2 (2)	O1—C19—O2	122.1 (3)
C4—C5—C6	108.2 (2)	O1—C19—C1'	125.5 (3)
C4—C5—C11	107.1 (2)	O2—C19—C1'	112.4 (3)
C6—C5—C11	106.03 (19)	O8—C20—H20A	109.5
C4—C5—H5	111.7	O8—C20—H20B	109.5
C6—C5—H5	111.7	H20A—C20—H20B	109.5
C11—C5—H5	111.7	O8—C20—H20C	109.5
C5—C6—C7	104.6 (2)	H20A—C20—H20C	109.5
C5—C6—H6A	110.8	H20B—C20—H20C	109.5
C7—C6—H6A	110.8	O6—C21—H21A	109.5
C5—C6—H6B	110.8	O6—C21—H21B	109.5
C7—C6—H6B	110.8	H21A—C21—H21B	109.5
H6A—C6—H6B	108.9	O6—C21—H21C	109.5
C8—C7—C6	110.8 (2)	H21A—C21—H21C	109.5
C8—C7—C17	111.3 (2)	H21B—C21—H21C	109.5
C6—C7—C17	103.5 (2)	O7'—C22'—H22D	109.5
C8—C7—H7	110.4	O7'—C22'—H22E	109.5
C6—C7—H7	110.4	H22D—C22'—H22E	109.5
C17—C7—H7	110.4	O7'—C22'—H22F	109.5
O5—C8—C7	106.0 (2)	H22D—C22'—H22F	109.5
O5—C8—C15	107.5 (2)	H22E—C22'—H22F	109.5
C7—C8—C15	111.7 (2)	N2—C23—C24	112.3 (3)
O5—C8—C9	108.5 (2)	N2—C23—H23A	109.1
C7—C8—C9	109.8 (2)	C24—C23—H23A	109.1
C15—C8—C9	113.1 (2)	N2—C23—H23B	109.1
O4—C9—C14	110.7 (2)	C24—C23—H23B	109.1
O4—C9—C10	107.86 (19)	H23A—C23—H23B	107.9
C14—C9—C10	103.6 (2)	C23—C24—H24A	109.5
O4—C9—C8	112.5 (2)	C23—C24—H24B	109.5
C14—C9—C8	109.5 (2)	H24A—C24—H24B	109.5
C10—C9—C8	112.3 (2)	C23—C24—H24C	109.5
O3—C10—C9	106.68 (19)	H24A—C24—H24C	109.5
O3—C10—C12	107.6 (2)	H24B—C24—H24C	109.5
C9—C10—C12	102.42 (19)	C6'—C1'—C2'	119.1 (3)
O3—C10—C11	107.93 (19)	C6'—C1'—C19	120.6 (3)
C9—C10—C11	117.1 (2)	C2'—C1'—C19	120.3 (3)
C12—C10—C11	114.4 (2)	N1—C2'—C3'	120.7 (3)
C17—C11—C1	119.2 (2)	N1—C2'—C1'	121.8 (3)
C17—C11—C5	97.83 (19)	C3'—C2'—C1'	117.4 (3)
C1—C11—C5	111.34 (19)	C4'—C3'—C2'	122.2 (3)
C17—C11—C10	107.79 (19)	C4'—C3'—H3'	118.9
C1—C11—C10	108.1 (2)	C2'—C3'—H3'	118.9
C5—C11—C10	112.4 (2)	C3'—C4'—C5'	120.8 (3)
C13—C12—C10	107.7 (2)	C3'—C4'—H4'	119.6
C13—C12—H12A	110.2	C5'—C4'—H4'	119.6
C10—C12—H12A	110.2	C6'—C5'—C4'	118.8 (3)
C13—C12—H12B	110.2	C6'—C5'—H5'	120.6
C10—C12—H12B	110.2	C4'—C5'—H5'	120.6
H12A—C12—H12B	108.5	C5'—C6'—C1'	121.7 (3)
C16—C13—C14	111.9 (3)	C5'—C6'—H6'	119.2
C16—C13—C12	110.8 (2)	C1'—C6'—H6'	119.2
C14—C13—C12	101.2 (2)	C2'—N1—H1A	120.0
C16—C13—H13	110.9	C2'—N1—H1B	120.0
C14—C13—H13	110.9	H1A—N1—H1B	120.0
C12—C13—H13	110.9	C18—N2—C23	110.7 (2)
O6—C14—C13	118.6 (2)	C18—N2—C17	115.3 (2)
O6—C14—C9	109.4 (2)	C23—N2—C17	113.2 (2)
C13—C14—C9	101.4 (2)	C19—O2—C4	121.5 (2)
O6—C14—H14	109.0	C10—O3—H3	109.5
C13—C14—H14	109.0	C9—O4—H4	109.5
C9—C14—H14	109.0	C8—O5—H5A	109.5
C16—C15—C8	119.7 (2)	C14—O6—C21	113.5 (3)
C16—C15—H15A	107.4	C22—O7—C16	110.3 (13)
C8—C15—H15A	107.4	C22'—O7'—C16	142 (2)
C16—C15—H15B	107.4	C20—O8—C1	113.5 (2)
			
O8—C1—C2—C3	172.1 (2)	O4—C9—C14—O6	70.7 (3)
C11—C1—C2—C3	46.5 (3)	C10—C9—C14—O6	−173.9 (2)
C1—C2—C3—C4	−54.4 (3)	C8—C9—C14—O6	−53.9 (3)
C2—C3—C4—O2	−170.1 (2)	O4—C9—C14—C13	−163.2 (2)
C2—C3—C4—C5	66.6 (3)	C10—C9—C14—C13	−47.9 (2)
C2—C3—C4—C18	−57.7 (3)	C8—C9—C14—C13	72.2 (3)
O2—C4—C5—C6	58.3 (2)	O5—C8—C15—C16	−136.5 (3)
C3—C4—C5—C6	−178.2 (2)	C7—C8—C15—C16	107.7 (3)
C18—C4—C5—C6	−51.7 (3)	C9—C8—C15—C16	−16.8 (4)
O2—C4—C5—C11	172.24 (18)	C8—C15—C16—O7'	139.0 (12)
C3—C4—C5—C11	−64.2 (3)	C8—C15—C16—O7	137.6 (8)
C18—C4—C5—C11	62.2 (3)	C8—C15—C16—C13	17.7 (4)
C4—C5—C6—C7	100.5 (2)	C14—C13—C16—O7'	−100.8 (8)
C11—C5—C6—C7	−14.2 (3)	C12—C13—C16—O7'	147.2 (8)
C5—C6—C7—C8	101.8 (2)	C14—C13—C16—O7	−84.6 (6)
C5—C6—C7—C17	−17.6 (2)	C12—C13—C16—O7	163.3 (6)
C6—C7—C8—O5	64.6 (3)	C14—C13—C16—C15	28.1 (3)
C17—C7—C8—O5	179.14 (19)	C12—C13—C16—C15	−83.9 (3)
C6—C7—C8—C15	−178.7 (2)	C1—C11—C17—N2	−48.0 (3)
C17—C7—C8—C15	−64.1 (3)	C5—C11—C17—N2	71.9 (2)
C6—C7—C8—C9	−52.4 (3)	C10—C11—C17—N2	−171.5 (2)
C17—C7—C8—C9	62.2 (3)	C1—C11—C17—C7	−170.4 (2)
O5—C8—C9—O4	−33.9 (3)	C5—C11—C17—C7	−50.5 (2)
C7—C8—C9—O4	81.5 (3)	C10—C11—C17—C7	66.1 (2)
C15—C8—C9—O4	−153.0 (2)	C8—C7—C17—N2	166.20 (19)
O5—C8—C9—C14	89.7 (2)	C6—C7—C17—N2	−74.8 (2)
C7—C8—C9—C14	−155.0 (2)	C8—C7—C17—C11	−75.4 (2)
C15—C8—C9—C14	−29.4 (3)	C6—C7—C17—C11	43.5 (2)
O5—C8—C9—C10	−155.8 (2)	O2—C4—C18—N2	−157.5 (2)
C7—C8—C9—C10	−40.4 (3)	C3—C4—C18—N2	84.4 (3)
C15—C8—C9—C10	85.1 (3)	C5—C4—C18—N2	−41.5 (3)
O4—C9—C10—O3	34.5 (3)	O1—C19—C1'—C6'	−176.2 (3)
C14—C9—C10—O3	−82.8 (2)	O2—C19—C1'—C6'	4.5 (4)
C8—C9—C10—O3	159.04 (19)	O1—C19—C1'—C2'	2.0 (5)
O4—C9—C10—C12	147.5 (2)	O2—C19—C1'—C2'	−177.3 (3)
C14—C9—C10—C12	30.1 (2)	C6'—C1'—C2'—N1	177.9 (3)
C8—C9—C10—C12	−88.0 (2)	C19—C1'—C2'—N1	−0.3 (5)
O4—C9—C10—C11	−86.4 (2)	C6'—C1'—C2'—C3'	−0.7 (4)
C14—C9—C10—C11	156.2 (2)	C19—C1'—C2'—C3'	−178.9 (3)
C8—C9—C10—C11	38.1 (3)	N1—C2'—C3'—C4'	−177.7 (3)
O8—C1—C11—C17	−55.8 (3)	C1'—C2'—C3'—C4'	0.9 (5)
C2—C1—C11—C17	68.0 (3)	C2'—C3'—C4'—C5'	−0.2 (5)
O8—C1—C11—C5	−168.5 (2)	C3'—C4'—C5'—C6'	−0.8 (5)
C2—C1—C11—C5	−44.7 (3)	C4'—C5'—C6'—C1'	0.9 (5)
O8—C1—C11—C10	67.6 (3)	C2'—C1'—C6'—C5'	−0.2 (5)
C2—C1—C11—C10	−168.6 (2)	C19—C1'—C6'—C5'	178.0 (3)
C4—C5—C11—C17	−75.3 (2)	C4—C18—N2—C23	169.7 (2)
C6—C5—C11—C17	40.1 (2)	C4—C18—N2—C17	39.6 (3)
C4—C5—C11—C1	50.3 (3)	C24—C23—N2—C18	79.2 (3)
C6—C5—C11—C1	165.7 (2)	C24—C23—N2—C17	−149.5 (3)
C4—C5—C11—C10	171.7 (2)	C11—C17—N2—C18	−57.3 (3)
C6—C5—C11—C10	−72.9 (2)	C7—C17—N2—C18	56.4 (3)
O3—C10—C11—C17	−173.14 (18)	C11—C17—N2—C23	173.8 (2)
C9—C10—C11—C17	−52.8 (3)	C7—C17—N2—C23	−72.5 (3)
C12—C10—C11—C17	67.1 (3)	O1—C19—O2—C4	11.8 (4)
O3—C10—C11—C1	56.8 (2)	C1'—C19—O2—C4	−168.9 (2)
C9—C10—C11—C1	177.13 (19)	C3—C4—O2—C19	−67.1 (3)
C12—C10—C11—C1	−63.0 (2)	C5—C4—O2—C19	57.5 (3)
O3—C10—C11—C5	−66.5 (2)	C18—C4—O2—C19	172.8 (2)
C9—C10—C11—C5	53.9 (3)	C13—C14—O6—C21	74.1 (3)
C12—C10—C11—C5	173.8 (2)	C9—C14—O6—C21	−170.5 (2)
O3—C10—C12—C13	110.3 (2)	O7'—C16—O7—C22	−78 (5)
C9—C10—C12—C13	−1.9 (2)	C15—C16—O7—C22	98.3 (9)
C11—C10—C12—C13	−129.7 (2)	C13—C16—O7—C22	−140.7 (8)
C10—C12—C13—C16	92.0 (3)	O7—C16—O7'—C22'	135 (8)
C10—C12—C13—C14	−26.7 (3)	C15—C16—O7'—C22'	130 (3)
C16—C13—C14—O6	46.8 (3)	C13—C16—O7'—C22'	−103 (3)
C12—C13—C14—O6	164.9 (2)	C2—C1—O8—C20	83.5 (3)
C16—C13—C14—C9	−72.9 (3)	C11—C1—O8—C20	−147.4 (3)
C12—C13—C14—C9	45.1 (3)		

### Hydrogen-bond geometry (Å, º)

**Table:**

*D*—H···*A*	*D*—H	H···*A*	*D*···*A*	*D*—H···*A*
O5—H5*A*···O6	0.84	2.34	2.909 (3)	126
O4—H4···O5	0.84	2.27	2.684 (3)	111
O4—H4···O3^i^	0.84	1.94	2.713 (3)	153
O3—H3···O4	0.84	1.99	2.524 (3)	121
N1—H1*B*···O1	0.88	2.00	2.666 (4)	131
N1—H1*A*···O8^ii^	0.88	2.19	2.999 (3)	152

Symmetry codes: (i) *x*+1/2, −*y*+1/2, −*z*+2; (ii) −*x*+3/2, −*y*, *z*+1/2.
