# Supplementary figures and images for: Crystal structure of sepaconitine, a C19-diterpenoid alkaloid from the roots of Aconitum sinomontanum Nakai
Source: Acta Crystallogr E Crystallogr Commun. 2015 Jul 8;71(Pt 8):o550–1. doi: 10.1107/S205698901501258X (PMC4571391; doi:10.1107/S205698901501258X)

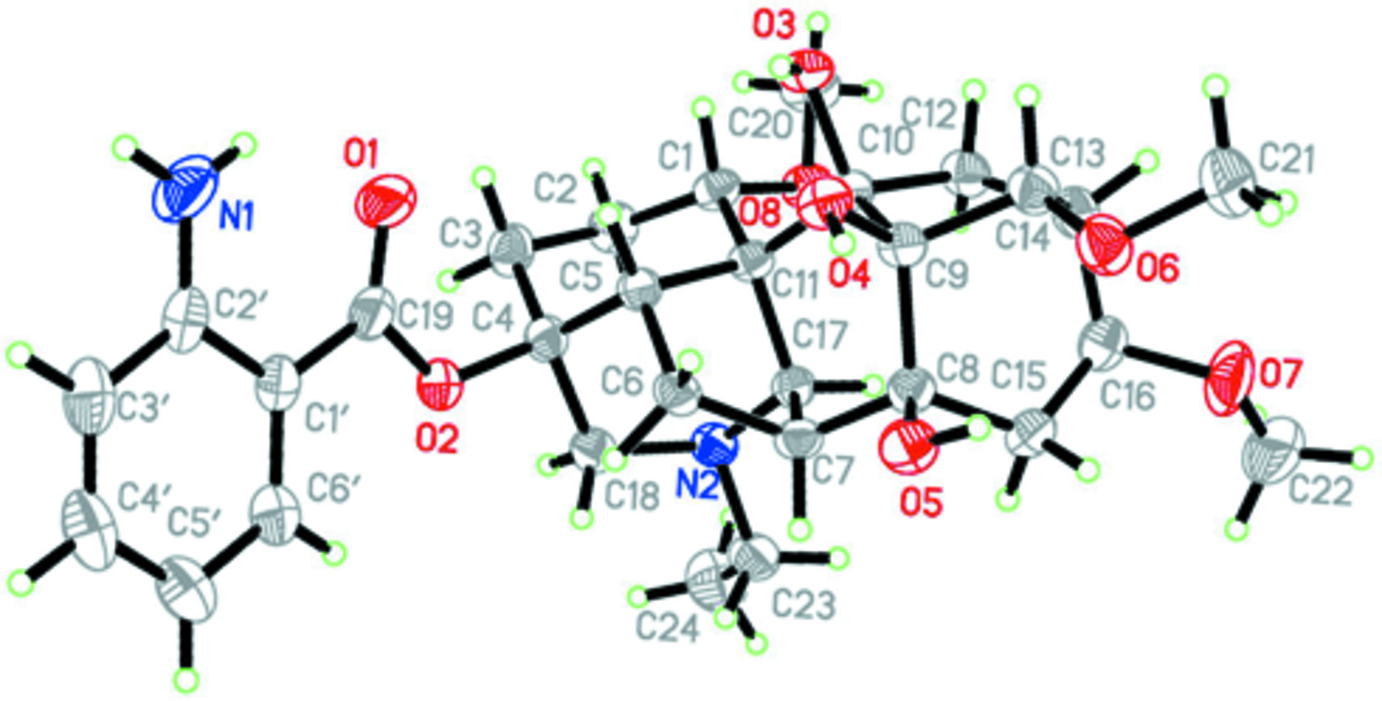

Supplement: Supplementary file 4 [file e-71-0o550-fig1.tif]

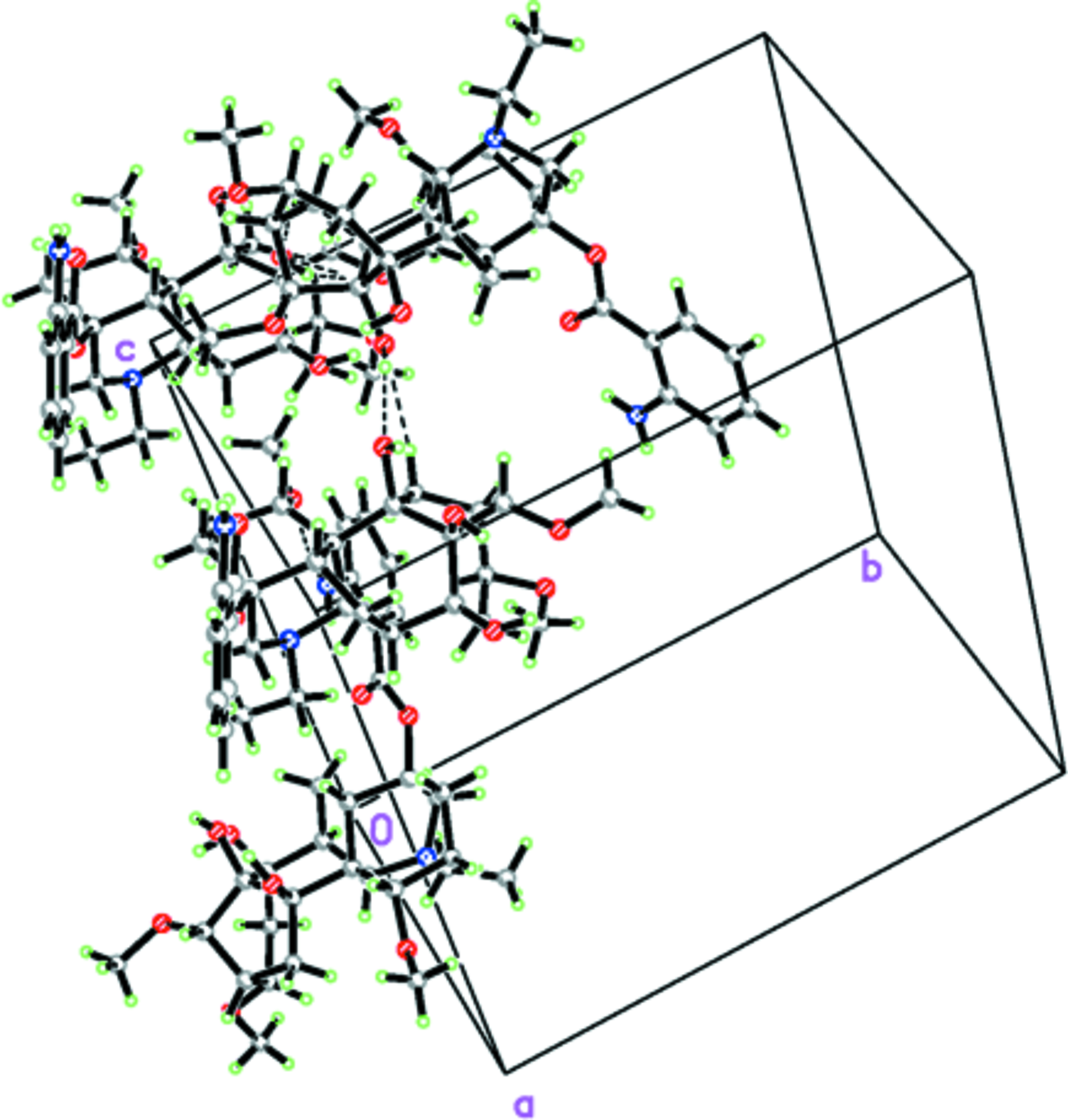

Supplement: Supplementary file 5 [file e-71-0o550-fig2.tif]
